# Analysis of Intraoperative Complications and Outcomes Associated With Obesity During Elective Laparoscopic Cholecystectomy

**DOI:** 10.1002/wjs.70470

**Published:** 2026-06-21

**Authors:** Thomas Coates, Marek Bak, Ashray Rajagopalan, Filip Pesevski, Anjana Ravi, Daniel Croagh, Geraldine Ooi

**Affiliations:** ^1^ Department of Surgery School of Clinical Sciences Monash University Clayton Victoria Australia; ^2^ Upper Gastrointestinal Hepatopancreaticobiliary Unit Monash Health Clayton Victoria Australia

## Abstract

**Background:**

As obesity becomes more common, understanding its risks in routine operations is increasingly important. This study assessed the impact of obesity on intraoperative complications during elective laparoscopic cholecystectomy.

**Methods:**

Retrospective data on elective laparoscopic cholecystectomies across a single metropolitan health network were collected from July 2021‐June 2023. Patients were stratified into WHO‐defined obesity classes. Intraoperative complication (measured by ClassIntra classification), intraoperative and postoperative outcomes were compared.

**Results:**

There were 713 patients included. Intraoperative complications graded as ClassIntra 1 and 2 were 11.0% and 6.1% for non‐overweight patients and rose to 35.3% and 13.2% respectively for class III obesity (*p* < 0.001). Increasing obesity class was independently associated with higher intraoperative complication severity, with a 2.59‐fold increase in the odds of a more severe complication category per class increase (*p* < 0.001). Obesity was associated with increased operative duration (*p* = 0.006) but did not increase the risk of postoperative morbidity (*p* = 0.88).

**Conclusion:**

Obesity significantly increases the risks of intraoperative complications in patients undergoing elective cholecystectomy, as measured by the ClassIntra classification, and contributes to increased operative duration. Despite this, postoperative morbidity remains low with no significant difference in length of stay, complications or readmissions across obesity classes.

## Graphical abstract

As obesity becomes more common, understanding its risks in routine operations is increasingly important. This study assessed the impact of obesity on intraoperative complications during elective laparoscopic cholecystectomy. Obesity significantly increases the risks of intraoperative complications in patients undergoing elective cholecystectomy, as measured by the ClassIntra classification, and contributes to increased operative duration. Despite this, postoperative morbidity remains low with no significant difference in length of stay, complications or readmissions across obesity classes.

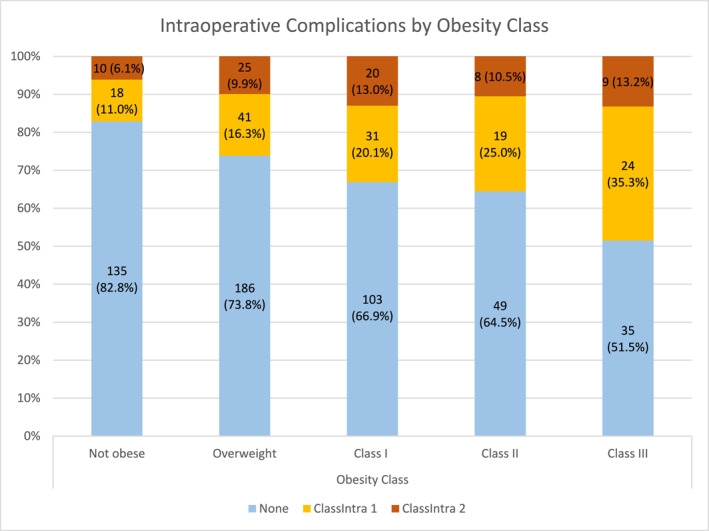

## 1 Introduction

Obesity is an increasingly common medical condition in Australia, with 66% of Australian adults being considered overweight or obese in 2022, an increase from 57% in 1995 [1, 2]. There is a well‐established relationship between obesity and postoperative complications.

Obese patients undergoing laparoscopic cholecystectomy are more likely to suffer postoperative wound dehiscence and infections and have higher rates of conversion to open surgery and readmission after discharge [3, 4, 5, 6, 7]. Patients with BMI> 50 undergoing laparoscopic cholecystectomy also have high rates of Clavien‐Dindo IV complications [8].

However, there is currently limited evidence surrounding the impact of obesity on technical complexity of laparoscopic surgery and intraoperative complications. Visibility, safe laparoscopic entry, operative space, tissue mobility and fragility are all adversely impacted by increased intraabdominal adiposity and may impact on the safety and fluidity of an operation. In laparoscopic cholecystectomy, cephalad retraction of the liver is an important maneuver to splay the hepatocystic triangle for safe dissection to obtain the critical view of safety [9]. Obesity is related to liver fibrosis and stiffness which may make this maneuver more difficult and increase the risk of intraoperative complications [10].

Recently, the ClassIntra classification system has been validated for classifying intraoperative complications [11, 12], providing comprehensive and uniform definitions with high inter‐rater correlation [13, 14]. Complications range from grade 1 being a small deviation from ideal operative course, such as minor bleeding requiring electrocautery, to grade 4 involving complications that threaten life or cause permanent disability (Table 1). This system allows us to take an objective approach to understanding intraoperative complications.

**TABLE 1:** ClassIntra classification of intraoperative complications with examples in laparoscopic cholecystectomy—adapted from Dell‐Kuster et al. [11] and Xu et al. [25].

Grade 0	Ideal operative course
Grade 1	Deviation from the ideal operative course without the need for additional intervention. May result in no or mild symptoms
Grade 1 example	Bleeding from gallbladder fossa or small liver capsule tear requiring pressure or cautery to control Spillage of bile or stones requiring washout or manual stone retrieval Intraoperative episodes of bradycardia or arrhythmia not requiring intervention
Grade 2	Deviation from the ideal operative course with the need for additional treatment or minor intervention. May result in moderate symptoms, not resulting in permanent disability or threat to life.
Grade 2 example	Bleeding from liver capsule tear, gallbladder fossa or cystic artery requiring additional clipping, ligation or use of hemostatic agents Diathermy injury to the liver or skin requiring observation or non‐procedural management Anesthetic events such as bronchospasm, hypotension or tachycardia requiring medication without persistent hemodynamic effect.
Grade 3	Deviation from the ideal intraoperative course with the need for additional moderate treatment or intervention. May result in severe symptoms, potentially leading to permanent disability and potentially life threatening.
Grade 3 example	Bleeding from liver, gallbladder fossa, cystic artery resulting in hemodynamic instability and requiring ligation, clipping, suture or administration of blood products Suspected bile ductal injury requiring abandonment of planned procedure, placement of a drain and/or postoperative imaging but no intervention Damage to bowel on port entry requiring resection or full thickness suture repair
Grade 4	Deviation from the ideal intraoperative course with the need for major and urgent treatment or intervention. Life threatening or leading to permanent disability.
Grade 4 example	Significant bleeding from cystic artery or liver resulting in massive transfusion, admission to intensive care unit or partial hepatectomy Injury to the common bile duct or duodenum requiring reconstructive surgery Cardiovascular collapse requiring defibrillation, cardioversion, admission to the intensive care unit
Grade 5	Death

The aim of the study was therefore to characterize the impact of obesity on intraoperative complications for patients undergoing elective cholecystectomy for cholelithiasis or related disease, utilizing the ClassIntra classification system.

## 2 Methods

### 2.1 Patient Cohort

This was a retrospective cohort study of all consecutive patients who underwent elective laparoscopic cholecystectomy at an Australian tertiary health network from July 2021 to June 2023. Patients were identified from operative records. Patients were eligible for inclusion if they were aged over 18 years and underwent elective cholecystectomy for cholelithiasis and related disease. Exclusion criteria were emergency operations, polyp or malignancy as an indication for surgery, history of prior cholecystostomy tube or upper abdominal surgery, or requirement for planned or unplanned common bile duct exploration.

### 2.2 Ethics Approval

This study was registered institutionally as a Quality Assurance activity and exempted from formal ethics approval.

### 2.3 Data Collection

Data were collected from electronic medical records. Intraoperative complications were graded from operation reports and anesthetic records according to the ClassIntra classification system by two independent reviewers. Disagreements were resolved by consensus of the reviewers. Other fields collected included patient height, weight and BMI, conversion to subtotal cholecystectomy, conversion to open surgery, duration of operation, hospital length of stay, Intensive Care Unit (ICU) admission data, length of stay and any postoperative complications graded per the Clavien‐Dindo II classification system (CD) [15]. Obesity was classified based on the World Health Organization definition (BMI< 25: not‐overweight, BMI 25–29.9: overweight, BMI 30–34.9: class I obesity, BMI > 35–39.9: class II obesity, BMI > 40: class III obesity) [16].

### 2.4 Statistical Analysis

De‐identified data was stored in a password protected online database and analyzed using R version 4.3.3. Our primary outcome was the relationship between ClassIntra complications and BMI class. Our secondary outcomes were operation duration, conversion to subtotal or open cholecystectomy, length of stay, Clavien‐Dindo complications, readmission within 30 days, return to theater and death. The Shapiro‐Wilks test was used to identify the distribution of data. Mean and standard deviation were calculated for normally distributed data, while median and interquartile range (IQR) were calculated for skewed data. Chi‐square goodness‐of‐fit test was used to analyze expected and observed outcomes and Chi‐square test of independence for frequency tables with Fisher's Exact Test used when expected cell size was under 5, Cochran‐Armitage test was used to assess linear trends across ordinal groups. For continuous data compared across multiple ordinal categories, Analysis of Variance (ANOVA) was used for parametric data and Kruskal‐Wallis for nonparametric data. Multivariate analysis was performed, with ordinal logistic regression used when the outcome variable was ordinal, logistic regression when the outcome was binary and linear regression when the outcome was continuous. A *p* < 0.05 was considered statistically significant.

## 3 Results

### 3.1 Patients

A total of 757 patients were identified of which 713 (94.2%) patients were included for analysis, 30 (4.0%) and 5 (0.7%) were excluded for polyp and malignancy as an indication respectively and 9 (1.1%) due to prior cholecystostomy or significant upper abdominal surgery. There were 163 (22.9%) not‐overweight patients, 252 (35.3%) overweight patients, 154 (21.6%) class I obesity, 76 (10.7%) class II obesity and 68 (9.5%) class III obesity patients.

Pre‐operative demographic data is shown in Table 2. Median age across BMI classes was broadly similar, though patients in Class II and Class III obesity tended to be younger than the overall cohort median of 53.5 years (IQR 40.2–63.5). Males comprised 32.7% of the overall cohort, with the highest proportion observed in the overweight group (42.9%). American Society of Anesthesiologists (ASA) grade increased with obesity severity, with ASA ≥ 3 present in 15.9% of not overweight patients compared with 64.7% of Class III obesity patients.

**TABLE 2:** Preoperative demographic data by obesity class.

		Total	Not overweight	Overweight	Class I obesity	Class II obesity	Class III obesity	*p* value
n =		713	163	252	154	76	68	
Age		53.5 (40.2–63.5)	51.6 (39.9–67.6)	54.9 (39.9–67.6)	54.9 (38.8–66.2)	46.9 (37.6–60.0)	48.4 (34.1–58.6)	0.020
Sex (male)		233 (32.7%)	47 (28.8%)	108 (42.9%)	46 (29.9%)	18 (23.7%)	14 (20.6%)	< 0.001
ASA	1	123 (17.3%)	41 (25.2%)	58 (23.0%)	17 (11.0%)	5 (6.6%)	2 (2.9%)	< 0.001
	2	392 (55.0%)	96 (58.9%)	135 (53.6%)	91 (59.1%)	49 (64.5%)	21 (30.9%)	0.050
	3	187 (26.2%)	25 (15.3%)	58 (23.0%)	43 (27.9%)	20 (26.3%)	41 (60.3%)	< 0.005
	4	11 (1.5%)	1 (0.6%)	1 (0.4%)	3 (1.9%)	2 (2.6%)	4 (5.9%)	0.02

*Note:* Represented as mean +/− standard deviation, median (interquartile range) or number (percentage).

Abbreviation: ASA, American Society of Anesthesiologist physical status classification system.

### 3.2 Primary Outcome

**FIGURE 1:**
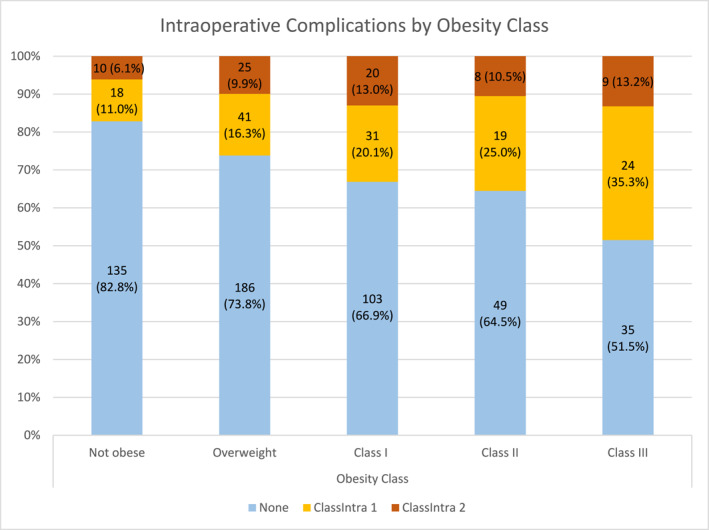
Intraoperative ClassIntra complications by obesity class.

Class 1 and 2 complications occurred in 133 (18.7%) and 72 (10.1%) patients respectively. There were no ClassIntra 3,4 or 5 complication. There was a significant relationship between BMI class and intraoperative complications as defined by the ClassIntra system (Table 3, Figure 1). As obesity increased from non‐overweight to class III obesity, risk of experiencing any intraoperative complication increased from 17.1% to 48.5% (*X* ^2^ = 26.7, *p* < 0.001). This trend was most pronounced in ClassIntra 1 complications increasing from 11.0% to 35.3% in non‐overweight to class III obesity groups (*p* = 0.001).

**TABLE 3:** Intraoperative and postoperative outcomes by obesity class.

		Total	Non‐overweight	Overweight	Class I obesity	Class II obesity	Class III obesity	*p*‐value
*n* =		713	163	252	154	76	68	
Intraoperative complications (ClassIntra)	None	508 (71.2%)	135 (82.8%)	186 (73.8%)	103 (66.9%)	49 (64.5%)	35 (51.5%)	0.094
	Class 1	133 (18.7%)	18 (11.0%)	41 (16.3%)	31 (20.1%)	19 (25.0%)	24 (35.3%)	0.001
	Class 2	72 (10.1%)	10 (6.1%)	25 (9.92%)	20 (13.0%)	8 (10.5%)	9 (13.2%)	0.34
	Cumulative risk	205 (28.8%)	28 (17.1%)	66 (26.2%)	51 (33.1%)	27 (35.5%)	33 (48.5%)	< 0.001
Operative time (minutes)		69 (56–88)	66 (55–80.5)	68 (53.5–88)	70 (58–89.3)	75 (59.8–93.3)	78 (58.8–104.0)	0.006
Approach	Open	3 (0.4%)	0	1 (0.4%)	0	1 (1.3%)	1 (1.5%)	0.19
	Subtotal	17 (2.4%)	4 (2.5%)	5 (2.0%)	5 (3.2%)	2 (2.6%)	1 (1.5%)	0.91
CD complications	CD I	40 (5.6%)	13 (8.1%)	15 (6.0%)	5 (3.2%)	4 (5.3%)	3 (4.4%)	0.48
	CD II	28 (3.9%)	5 (3.0%)	10 (4.0%)	7 (4.5%)	2 (2.6%)	4 (5.9%)	0.83
	CD IIIa	3 (0.4%)	1 (0.6%)	0	0	1 (1.3%)	1 (1.5%)	0.12
	CD IIIb	9 (1.3%)	3 (1.8%)	1 (0.4%)	3 (1.9%)	2 (2.6%)	0	0.26
	CD IVb	2 (0.3%)	0	2 (0.8%)	0	0	0	0.69
	Cumulative risk	83 (11.5%)	22 (13.5%)	28 (11.1%)	15 (9.7%)	9 (11.8%)	8 (11.8%)	0.88
Length of stay (hours)		27.7 (24.8–32.3)	27.6 (24.7–31.1)	27.5 (24.5–33.5)	27.8 (25.0–33.5)	27.6 (25.6–37.4)	28.0 (25.7–31.5)	0.96
Readmission		21 (2.9%)	6 (3.7%)	8 (3.2%)	4 (2.6%)	2 (2.6%)	1 (1.5%)	0.97
Return to theater		8 (1.1%)	3 (1.8%)	2 (0.8%)	2 (1.3%)	0	0	0.72

*Note:* Represented as mean +/− standard deviation, median (interquartile range) or number (percentage).

Abbreviation: CD, Clavien‐Dindo postoperative complication grade.

Multivariate analysis showed obesity class, age, sex (male) and operation length were independently associated with intraoperative complications (Table 4). ASA was excluded as a covariate due to minimal variability in higher obesity classes, inclusion resulted in similar coefficients but unstable results. Ordinal logistic regression showed increasing obesity class was independently associated with higher intraoperative complication severity, with a 2.59‐fold increase in the odds of a more severe complication category per class increase (95% CI 1.65–4.08, *p* < 0.001). Increasing age and longer operation duration were also significantly associated with intraoperative complications, with odds ratio of 1.02 per year of age (95% CI 1.01–1.03, *p* = 0.02), and 1.02 per minute of operating (95% CI 1.01–1.03, *p* < 0.001).

**TABLE 4:** Multivariate analysis of operative factors.

	Coef	95% CI	*p*‐value
Intraoperative complications			
Obesity class	2.59	1.65–4.08	< 0.001
Age	1.02	1.01–1.03	0.0019
Sex (male)	1.44	0.99–2.06	0.050
Operation duration	1.02	1.01–1.03	< 0.001
Operation duration			
ClassIntra	20.86	16.10–25.63	< 0.001
Obesity class	12.45	7.07–17.83	< 0.001
Age	0.17	0.05–0.29	0.005
Sex (male)	7.69	3.38–12.00	< 0.001
Subtotal	28.65	15.81–41.90	< 0.001
Open	62.99	32.68–93.26	< 0.001

### 3.3 Secondary Outcomes

Operation duration was significantly longer in patients as obesity class rose (Table 3) with median duration of operation extending from 66 min for not‐overweight patients to 78 min for class III obesity patients (*p* = 0.006). In multivariate analysis this relationship held with patients in higher obesity classes showing a longer operative time by 12 min and 27 s compared to those in a lower class (*p* < 0.001) when controlling for intraoperative complications, age, sex and conversion to subtotal or open operations (Table 4). Conversion to subtotal cholecystectomy or open operation was uncommon (0.4%) and there was no significant difference between rates in each obesity class (*p* = 0.91, *p* = 0.19 respectively).

Clavien‐Dindo I and II complications represented the majority of recorded complications with an incidence of 5.6% and 3.9% respectively. There was no significant difference in postoperative morbidity in each obesity class (Table 3). There were no deaths. Median length of stay was 27.7 h with a strong positive skew suggesting most patients discharging without complication while a few experienced prolonged complex admissions. There were no differences in length of stay between obesity classes (*p* = 0.96). Few patients experienced readmission (*n* = 21, 2.9%) or return to theater (*n* = 8, 1.1%) within 30 days, and there was no significant difference between rates across obesity classes (*p* = 0.97 and *p* = 0.72) (Table 3).

In univariate and multivariate analysis, obesity did not affect postoperative complications, length of stay or risk of readmission. Age was significant associated with these outcomes, with an increase in each year of age increasing the risk of being in a higher CD complication group by 4%, length of stay by 0.57 h, readmission risk by 3% (*p* < 0.001, *p* < 0.001 and *p* = 0.04) (Table 5).

**TABLE 5:** Multivariate analysis of postoperative outcomes.

	Coef	95% CI	*p*‐value
Postoperative complications			
Obesity class	1.16	0.61–2.13	0.63
Age	1.04	1.03–1.06	< 0.001
Sex (male)	0.98	0.58–1.61	0.93
Length of stay			
Obesity class	1.24	−9.23‐19.70	0.82
Age	0.57	0.33–0.81	< 0.001
Sex (male)	5.86	−2.63‐14.35	0.18
Readmission			
Obesity class	0.64	0.09–2.27	0.55
Age	1.03	1.00–1.06	0.04
Sex (male)	1.61	0.64–3.98	0.30

## 4 Discussion

Planning is paramount to a safe and successful operation and having an objective understanding of the risk profile of a patient being worked up for an operation is a key component of this planning. In this study, we have provided objective evidence for the relationship between obesity and intraoperative complications in patients undergoing elective cholecystectomy. While this relationship has long been presupposed by surgeons, understanding the increasing risk across higher obesity classes provides useful insight into the difficulties that may be faced. This insight may better inform the decision to proceed with surgery, pre‐operative optimisation, and time and resource planning. Furthermore, it provides an important reference to the risks that must be discussed while obtaining informed consent from patients with obesity.

Both univariate and multivariate analyses showed that patients in higher obesity classes were significantly more likely to experience higher levels of intraoperative complications when compared to patients in lower obesity classes. We observed this trend despite higher obesity classes having a younger age and higher proportion of females, both protective factors (*p* < 0.001 and *p* = 0.050, Table 4). Higher obesity class was also related to increased operative time, again despite these cohorts having younger patients and more females, both protective factors (*p* = 0.005 and *p* < 0.001).

Prior studies have relied on major complications such as conversion to subtotal, open cholecystectomy, bile duct injuries or major blood loss as markers of intraoperative complications and consistently found increased operative times but limited or no evidence for increase in risk of other complications across obesity classes [5, 17, 18, 19, 20]. Our analysis using the ClassIntra system allows us to identify less severe but still clinically relevant complications and gain further insight into the true risks and challenges of obesity in laparoscopic cholecystectomy. Laparoscopic cholecystectomy is demonstrably more difficult in obese patients and related to higher rates of intraoperative complications. Importantly, our study shows that each obesity class carries more risk for intraoperative complications than the class below it.

Despite the increase in intraoperative complications, we found no difference in conversion to open procedure, subtotal cholecystectomy rates, or postoperative outcomes experienced between BMI classes. Limited evidence shows significant impact of obesity in postoperative outcomes for cholecystectomy patients in patients with BMI> 50 and showed higher rates of Clavien‐Dindo IV complications, longer lengths of stay and an increased risk of readmission [8, 18]. This has not been shown in our study, perhaps due to low overall rates of open and subtotal cholecystectomies and postoperative complications. It may also suggest that whilst we encounter intraoperative challenges associated with obesity, surgeons are able to identify and successfully manage these difficulties. This would be supported by the finding that patients who experienced intraoperative complications had operation duration 21 min longer (Table 4), even when controlling for other factors such as obesity, age, sex and subtotal or open cholecystectomy.

There is limited data on the routine use of preoperative weight loss programs to improve surgical safety for cohorts with obesity undergoing non‐bariatric procedures. Evidence shows safety and feasibility of very‐low calorie diets in preoperative planning [21, 22]. These studies did not show significant improvements in postoperative complications or outcomes [21, 22, 23] but were limited in study size and included patients with a mix of non‐bariatric operations. One study suggested a positive effect of pre‐operative weight loss on operative times [23], and therefore the possibility of treatment effect in larger cohorts. Mechanistically, very‐low calorie diets result in early reduction in liver size of up to 5%–20%, which may greatly assist access in cholecystectomy [24]. The effect of pre‐operative weight loss on intraoperative complications has not previously been reported in cholecystectomy.

Important limitations of our study include its retrospective nature, which may introduce observer or misclassification bias. We endeavored to minimize inaccuracies by using a consensus review process and complemented these data with objective postoperative outcome measures, however we had no direct method to verify the accuracy of operations reports completed at the time of surgery. It is possible that intra‐operative events may have been underreported and there may exist a bias to report only in patients who are already seen as higher risk. Our study was conducted with data from a single health network, and this may limit generalizability. Additionally, we excluded non‐cholelithiasis related indications for cholecystectomy, as well as patients undergoing emergency cholecystectomy, in order to exclude significant disease‐related confounding variables that would naturally increase intraoperative challenges. Therefore, this data is not applicable to emergency cases. Our study stratified obesity classes according to the WHO classification, however observations for patients with class III obesity were limited (*n* = 68) and our study may be underpowered to detect small but clinically relevant complications. Our study did not further stratify high risk obesity patients to detect differences in outcomes in patients with very high BMI due to these limited numbers.

This is the first study to use the newly validated ClassIntra system to investigate the relationship between obesity and intraoperative complications. It shows a clear relationship between obesity, intraoperative complications and operative duration. Future research should explore the relationship between obesity and other intra‐abdominal laparoscopic surgeries, as well as extend the scope to emergency operations. An opportunity exists for further research looking at the impact of weight loss programs prior to cholecystectomy and other non‐bariatric abdominal procedures on intraoperative and postoperative outcomes, particularly overweight patients where our study suggests the most significant potential benefit lies.

## Author Contributions

**Thomas Coates:** investigation, writing – original draft, methodology, software, formal analysis, writing – review and editing, data curation, validation, visualization. **Marek Bak:** conceptualization, investigation, writing – review and editing, data curation, methodology, writing – original draft, validation. **Ashray Rajagopalan:** conceptualization, investigation, writing – review and editing, methodology, data curation. **Filip Pesevski:** validation, writing – review and editing, data curation, writing – original draft. **Anjana Ravi:** validation, writing – review and editing, data curation, writing – original draft. **Daniel Croagh:** conceptualization, investigation, methodology, supervision, project administration, writing – review and editing. **Geraldine Ooi:** conceptualization, investigation, writing – review and editing, project administration, supervision, resources, validation, visualization.

## Funding

The authors have nothing to report.

## Conflicts of Interest

The authors declare no conflicts of interest.

## Data Availability

The data that support the findings of this study are available on request from the corresponding author. The data are not publicly available due to privacy or ethical restrictions.
